# Evaluating the Functional Characteristics of Certain Insect Flours (Non-Defatted/Defatted Flour) and Their Protein Preparations

**DOI:** 10.3390/molecules27196339

**Published:** 2022-09-26

**Authors:** Ewelina Zielińska

**Affiliations:** Department of Analysis and Evaluation of Food Quality, University of Life Sciences in Lublin, Skromna Street 8, 20-704 Lublin, Poland; ewelina.zielinska@up.lublin.pl

**Keywords:** edible insects, entomophagy, functional properties

## Abstract

Edible insects as an alternative source of protein are gaining increasing attention, leading to new opportunities for their use in food processing. In this study, the functional properties, such as water and oil holding capacity, foaming, and emulsifying properties, of the most popular insect forms (flour, defatted flour, and protein preparations), such as *Gryllus asimillis*, *Acheta domesticus*, and *Zophobas morio*, were studied. Moreover, proximate analysis, protein extraction yield and efficiency, and sensory analysis, were evaluated. Defatting the flours yielded the highest protein content of all the insect forms tested, in the range of 70.51 to 76.02%, significantly reducing their calorific value by up to 35% for *Z. morio*. Generally, protein preparations exhibit the best functional properties among studied forms, and the most significant differences are noticeable in foaming capacity—near 30% higher than flours. Furthermore, all samples scored well in the sensory test (overall score 3.76–4.47) except for the *Z. morio* flour (2.93), which may exclude it from being used in the food industry. The results show that the insect forms studied, due to their good functional properties, can become a valuable component of food recipes, positively impacting the characteristics of the designed food.

## 1. Introduction

Edible insects have received global attention as a potential solution to the problem of protein deficiency, water shortage, and global warming due to the high animal-based protein food production [1]. Nevertheless, food neophobia is a major limitation that reduces the acceptability of this alternative food source [2,3]. Many studies have suggested that including edible insects in flour and protein concentrates or isolates in various food products can increase consumer acceptance [4,5,6]. Dried insects may be powdered, and raw or cooked insects may be ground or crushed, making them less recognizable to consumers. Because of their high protein and mineral content, insects can be used as an additive in conventional food production, and can significantly enrich the nutritional value of products [7]. Individual ingredients, such as protein or fat, can also be isolated from insects and used in food technology. However, the processes for extracting nutrients from insects are quite costly and, therefore, require ongoing development on an industrial scale to make them more cost-effective and applicable to the food production sector [8].

It is consequently necessary to study the nutritional and functional properties (e.g., solubility, foaming, gelation, and emulsions) of insect protein flours and concentrates to optimize the quality of the ingredients and to target the use of particular insect species. Many researchers have already addressed this issue, and studies about the functional properties of several insect species were made [9,10,11,12]. However, more than 2100 insect species have been documented in the literature as edible, and the characteristics of each species must be considered separately [13]. These properties could be helpful to clarify the use of insect powder or protein extracts in different food products. Moreover, obtaining a wide range of information on an insect species is essential for it to be recognized as a novel food by the European Commission [14].

The easiest and most acceptable way to use insects is to powder them [15,16]. The preparation of flours could also be extended to remove chitin, which may reduce the digestibility of proteins or the absorption of minerals. Once obtained, insect flour can be used directly as an ingredient in food preparations or to produce insect protein concentrates or isolates [17]. In turn, defatting is one of the most effective processes for increasing the protein content by reducing the lipid content of raw materials, making it an obvious solution for processing insect flours [1]. Moreover, hexane is the most commonly used solvent for producing defatted insect flour and protein extracts for its high oil recovery, usually more than 96% [2,18,19].

Making isolates or protein concentrates from insects is another way to incorporate them into food formulations. Several authors have presented insect protein extraction methodologies to produce protein concentrates and isolates, similar to the plant protein extraction methods [11,17,20,21]. Nevertheless, current protein extraction methods limit their use due to low extraction yields or higher costs than in the case of the production of flours or defatted flours [17].

The techno-functional properties of high-protein ingredients, such as water-holding capacity (WHC) and oil-holding capacity (OHC), are essential attributes considered during food formulation. Foaming capacity (FC) and foam stability (FS), as well as emulsion activity (EC) and emulsion stability (ES), are equally important factors determining the applicability of an additive to a particular food production sector. The percentage of substitution of common ingredients and sensory attributes of final products must also be considered.

This study aimed to determine the functional properties of three species of insects (*Zophobas morio*, *Gryllus assimilis*, *Acheta domesticus*) prepared in three forms—flours, defatted flours, and protein preparations. Moreover, nutritional and sensory analysis of the studied forms was performed. Selected species of insects are popular to breed in Poland, as well as in the whole of Europe. Moreover, *Acheta domesticus* was reported to have the biggest potential to be used as food and feed in the European Union by EFSA [22].

## 2. Results and Discussion

### 2.1. Nutritive Value

The chemical composition of the studied samples is reported in Table 1. The defatted flours contained significant quantities of protein. Defatted flours from crickets were found to be the richest in protein—76.02 ± 0.53 for *Gryllus assimilis* and 75.35 ± 0.53 for *Acheta domesticus*, but the protein content was also high in defatted superworm flour—70.51 ± 0.49. Among the protein preparations, the highest protein content was recorded for crickets (64.00 ± 0.45—*Gryllus assimilis* and 66.34 ± 0.46—*Acheta domesticus*) due to the higher protein content of flours from these insects (59.24 ± 0.41 and 64.93 ± 0.45, respectively) than from superworm (49.06 ± 0.34). The protein content is most important, as it is responsible for the insect forms’ functional properties. However, functional properties can also be associated with other flours constituents, such as fat. Furthermore, fat interacts with hydrophobic amino acids changing their techno-functional properties, such as the oil-holding capacity and emulsifying properties [12]. The highest fat contents were found in the superworm forms studied (41.9 ± 1.53—flour, 6.51 ± 0.67—defatted flour, 23.22 ± 1.24—protein preparate). Among the crickets, *G. assimilis* had a higher fat content (26.25 ± 0.45) than *A. domesticus* (18.54 ± 0.34), but its content in defatted flours and protein preparations was not statistically significantly different between species (*p* < 0.05). A reduction in the fat content of defatted flours and protein preparations resulted in a concomitant increase in the carbohydrate content relative to insect flours (*p* < 0.05). The flours were found to be richest in energy value of the forms tested because the highest fat content characterized them.

### 2.2. Extraction Yield and Efficiency

**Figure 1:**
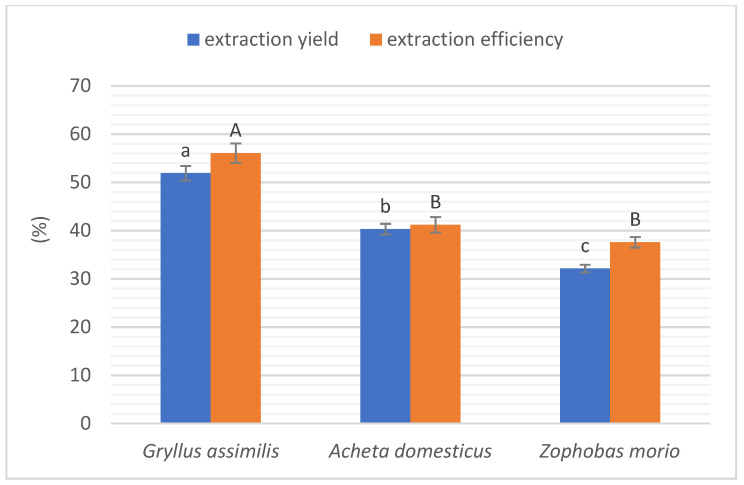
Extraction yield and efficiency of protein. Different letters indicate a significant difference (*p* < 0.05) for extraction yield. Different capital letters indicate a significant difference (*p* < 0.05) for extraction efficiency.

The yield and extraction efficiency of protein from insects are shown in Figure 1. The highest parameters were found for G. assimilis (51.9 and 56.07%, respectively). In the case of *A. domesticus*, these values were slightly lower (40.32 and 41.19%, respectively), whereas superworm showed even lower values (32.14 and 37.57%, respectively). The values of these indicators demonstrate that the type of extraction used is more efficient than water extraction. Chatsuwan et al. [23] determined the yield of two grasshopper species’ protein water extraction at 7.35 ± 0.19 and 7.49 ± 0.19%. Similarly, the protein yield in soluble locust fraction from *L. migratoria* was only 9.83% [24]. Despite a higher extraction efficiency than in the cited examples, the protein content in the obtained preparations was slightly higher than in insect flours (Table 1). In contrast, the defatted flours had the highest protein content, so choosing the most protein-rich insect form defatting the flour would probably be more economical than isolating the protein and, at the same time, more effective. However, protein content is not the only important issue in these insect forms. The advantage of defatted flours over protein preparations is the content of other valuable ingredients, such as minerals or vitamins. However, these two forms of insect will also differ in other properties, e.g., functional properties, which, in turn, will predispose their use in specific sectors of the food industry. This is where protein preparations may have an advantage, and why evaluating and comparing these properties is important.

### 2.3. Functional Properties

#### 2.3.1. Water and Oil Holding Capacity

Water holding capacity (WHC) and oil holding capacity (OHC) are considered key performance characteristics in food applications, especially in shaping food texture, and are significantly influenced by the composition of the formulations tested [25]. WHC is the ability of a protein matrix to retain as much water as possible per gram of sample material against gravity, regardless of whether it is bound or physically trapped water. OHC is the physical retention of oil and is related to taste and texture, both desirable properties for retaining flavor and tenderness [19].

**Figure 2:**
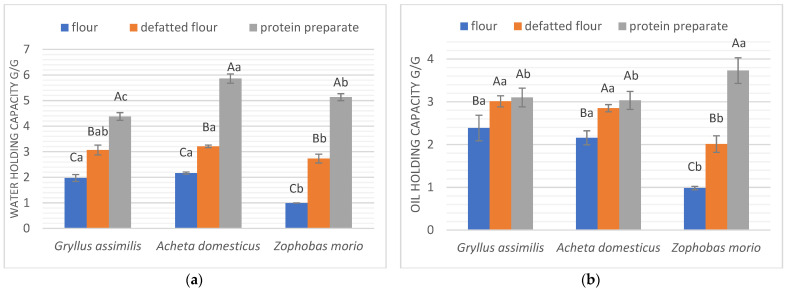
Absorbing properties of studied insect forms. (**a**) Water holding capacity (g/g); (**b**) oil holding capacity (g/g). Different letters in the same form of an insect (flour, defatted flour, protein preparation) indicate a significant difference (*p* < 0.05). Different capital letters in the same insect species indicate a significant difference (*p* < 0.05).

Figure 2 shows the water holding capacity (a) and oil holding capacity (b) of the studied insect forms. Generally, we observe a similar trend for all studied samples: the protein preparations have the highest water and oil holding capacities, whereas the lowest properties characterize flours. However, defatted flours and protein preparations derived from crickets are an exception—they have the same OHC (*p* < 0.05). All forms of *A. domesticus* were characterized by the highest WHC of the insect species tested: protein preparation—5.86 g/g, defatted flour—3.21 g/g, flour—2.16 g/g. The WHC of this cricket is higher than for soy isolate (4.47 g/g), where legumes are known for being high in protein with good functional properties [11]. The highest oil holding capacity was determined for the superworm protein preparation—3.73 g/g. Superworm also recorded the most significant difference between this parameter tested for flour (only 0.98 g/g) and the other forms. This is probably a result of the high fat content in superworm (41.9 ± 1.53). The OHC for all crickets tested forms ranged from 2.16 g/g for *A. domesticus* flour to 3.10 g/g for the *G. asimillis* protein preparation, and can be compared to the similarly determined OHC for silkworm (*Bombyx mori*) larvae and pupae (252.18% and 284.87%, respectively) [26], flours and protein preparation from *Tenebrio molitor* (1.71 and 2.74 g/g, respectively), *Gryllodes sigillatus* (2.82 and 3.33, respectively), and *Schistocerca gregaria* (1.98 and 3.22, respectively), [11] or legumes such as kidney bean flour (2.2–2.3 kg/kg) [27]. Other popular legumes used in food formulations, such as chickpea, lentil, and soy, were characterized by lower OHC than the studied insect forms, suggesting the possibility of substituting these materials in food products requiring high OHC values.

The significant differences in water and oil holding capacity between protein preparations and defatted flours, as well as insect flours, is a good indicator of the applications of these forms for different food products. For example, WHC is an important characteristic in the meat industry, in sausage formulation, and in the bakery industry, in bread and cake production [28]. This functional property is associated with improved texture and moisture content in foods. In turn, good OHC is required in food applications such as bakery products, ground meal formulation, and meat substitutes [11].

#### 2.3.2. Foaming Properties

The mechanism of foam formation involves the migration, unfolding, and reorganization of particles at the air–water interface to reduce surface tension [17]. Foam formation is dependent on several factors, including protein structure. Importantly, a good foaming capacity is not always correlated with a good stability of these foams, which is crucial in food formulation [19,29]. Therefore, the most desirable insect form will be one with a good foaming capacity and high foam stability.

**Figure 3:**
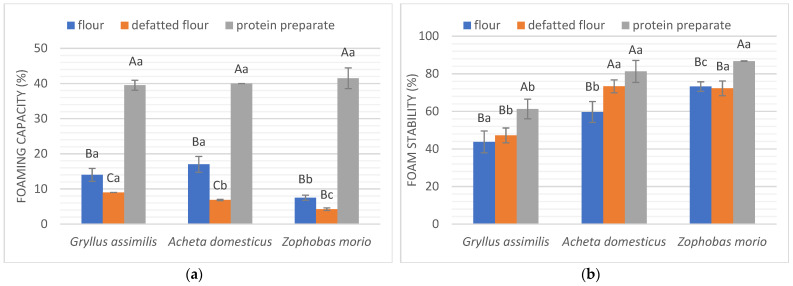
Foaming properties of studied insect forms. (**a**) Foaming capacity (%); (**b**) foam stability (%). Different letters in the same form of an insect (flour, defatted flour, protein preparation) indicate a significant difference (*p* < 0.05). Different capital letters in the same insect species indicate a significant difference (*p* < 0.05).

Figure 3 demonstrates the foaming capacity (a) and foam stability (b). All protein preparations were characterized by the highest foaming capacity at the same level, approximately 40% (*p* < 0.05). Among these, the preparations from *A. domesticus* and *Z. morio* had the highest foam stability (about 80%, *p* < 0.05), whereas the *G. assimilis* preparation exhibited 61.28% foam stability. The difference is significant and is not based on protein content because protein preparations did not contain the most protein. Some of the factors that affect good foaming properties are protein amphiphilicity and surface hydrophobicity, specifically the presence of thiol and hydrophobic groups of amino acids [17]. The highest properties can be correlated with the highest hydrophobic amino acid content in the protein of the studied forms. However, it may also depend on the location of hydrophobic amino acid residues on the protein surface [11]. A higher amount of hydrophobic amino acids in *Z. morio* (534 mg/g) and *A. domesticus* (532.2 mg/g) protein than in *G. assimilis* (445.8 mg/g) protein may be the reason for the better stability of foams created with them. These species were also characterized by higher cysteine content (7.6 mg/g and 8.3 mg/g, respectively) than *G. assimilis* (6.2 mg/g) [30]. Equally high differences in the foaming capacity between the flour and the protein preparation were recorded for the cricket, *Gryllodes sigillatus* [11]. Furthermore, the protein composition itself can affect the foaming capacity and stability. For example, the salt-soluble protein fraction of *T. molitor* demonstrated a higher foaming capacity than the water-soluble fractions [31]. Furthermore, the insect flours had a higher foaming capacity than the defatted flours, and those obtained from crickets had better properties than those from superworm.

In food technology, foams improve food’s texture, consistency, and appearance [17]. The most common example of a food group where foaming is an important functional characteristic is in the production of desserts and cocktails [17,28]. Currently, eggs are the most widely used foaming agent in food products, so protein preparations showing the best foaming properties may offer an alternative to them, and have the potential for such applications in food.

#### 2.3.3. Emulsifying Properties

Emulsions are homogeneous mixtures of two immiscible liquids, whether they are droplets of oil in water or droplets of water in oil. The formation and stabilization of food emulsions by reducing the surface tension at the oil–water interface are possible due to the amphiphilic nature of the proteins. This functional property has applications in many food industries, such as baked goods, mayonnaise, salad dressing, frozen desserts, and minced meats [19]. For example, good emulsion activity and stability are essential for the meat industry; to avoid water loss, sausage recipes use strong emulsifiers [17,28].

**Figure 4:**
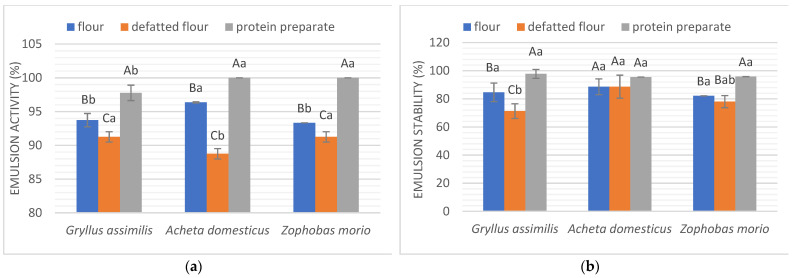
Emulsifying properties of studied insect forms. (**a**) Emulsion activity (%); (**b**) emulsion stability (%). Different letters in the same form of an insect (flour, defatted flour, protein preparation) indicate a significant difference (*p* < 0.05). Different capital letters in the same insect species indicate a significant difference (*p* < 0.05).

The results of the emulsion activity and emulsion stability are presented in Figure 4. The highest values of emulsion activity were observed for protein preparations from *A. domesticus* (100%) and *Z. morio* (100%). Only a slightly lower value was noted for the *G. assimilis* protein preparation (97.78%). Emulsions prepared with protein preparations also had the best stability, which varied from 95.45 to 97.78%. In turn, defatted flours had the lowest emulsion capacity, but the values were still high—near 90%. Among the insect flours obtained, *A. domesticus* was found to have the highest emulsion capacity value—96.36%. The emulsion stability for flours and defatted flours was at a similar level, except for *G. assimilis*, where the emulsion stability for defatted flour was statistically lower than non-defatted flour (*p* < 0.05).

Key factors for emulsion activity in the case of proteins are the ratio between hydrophilic and hydrophobic amino acids and the secondary structure of the protein. The exposure of hydrophobic amino acids after the denaturation of the protein allows them to interact with lipid molecules, increasing their emulsion activity [17]. We note an improvement in the emulsion activity for protein preparations relative to flours. It can be concluded that this is due to a change in the protein occurring during its extraction. The increment in emulsion activity could be due to the breakdown of large protein molecules and the exposure of the hydrophobic amino acids [12,17]. A comparison of three differently produced protein extracts from aphids using mass spectrometry and gel electrophoresis suggests that protein extraction methods affect the properties of the extracted proteins. In addition, another type of protein extraction had the unique ability to extract certain types of aphid proteins [32].

### 2.4. The Sensory Analysis

The addition of insects to food, whether in the form of flour or protein preparations, affects the functional properties of the food, but also the sensory properties. Color, consistency, smell, and overall acceptability were evaluated to assess the insect form’s sensory acceptability. The sensory analysis of edible insect flours, defatted flours, and protein preparations thereof is presented in Table 2. In the evaluation of color, smell, and overall acceptability, no significant statistical differences were found for all tested samples (*p* < 0.05). For consistency, defatted flours were characterized by better notes. It was noticeable that these samples were the most powdery and pleasant to the touch. In turn, the worst ratings were achieved by flour from *Z. morio*, probably due to its high fat content (41.9 ± 1.53%)—much more than in all other forms. The flour was sticky to the touch and had lumps in it. Due to this fact, the calculated overall score of the product was also the lowest—2.93 ± 0.59. In turn, the best rating was obtained for defatted flour from *A. domesticus*—4.47 ± 0.47. Generally, the ratings for the studied characteristics were relatively high. The score for color ranged from 3.13 ± 1.02 to 4.63 ± 0.5, the smell from 3.25 ± 0.77 to 4.38 ± 0.62, and consistency from 2.25 ± 0.68 to 4.63 ± 0.5. The overall acceptability varied from 2.69 ± 0.7 to 4.44 ± 0.63. These results make it possible to conclude that the insect forms studied can be accepted as ingredients in food products without negatively altering their sensory characteristics. The exception may be *Z. morio* flour, which can negatively modulate the sensory characteristics of food. Combined with its poor ability to absorb water and fat, we may consider this flour less valuable than the others.

## 3. Materials and Methods

### 3.1. Raw Materials

The superworms, *Zophobas morio* (Fabricius, Coleoptera: Tenebrionidae) (larvae); crickets, *Gryllus assimilis* (Fabricius, Orthoptera: Gryllidae) (adult); and crickets, *Acheta domesticus* (Linnaeus, Orthoptera: Gryllidae) (adult) were obtained from a commercial supplier from Poland. All individuals of these species were fasted for approximately 48 h to clear their gastrointestinal tract of any residual food. For each species tested, approximately 0.5 kg of material was frozen and lyophilized. Afterwards, the insects were ground in a laboratory grinder (IKA A11 basic) to obtain flour. The flour was passed through a 20-mesh sieve to obtain a uniform particle size.

### 3.2. Obtaining the Protein Preparation

The method of Girón-Calle, Alaiz, and Vioque [33] was slightly modified for protein isolation. Briefly, insect flour was stirred with 0.2% NaOH (pH 11) at a ratio of 1:10 (*w*/*v*), for 1 h at room temperature. Next, centrifugation at 8000 *g* and the precipitation of proteins at the isoelectric point (pH 4.5) were carried out. Finally, precipitated proteins were centrifuged for 20 min at 8000 *g* and washed with distilled water. Afterwards, the protein preparations were lyophilized and kept at −18 °C until further analysis.

### 3.3. Defatting of Flour

Fat removal from flours was carried out according to the methodology of Bußler et al. [12] with modifications. First, fat extraction with hexane was used—5 parts of hexane were used per 1 part of the flour, and then stirred with a magnetic stirrer for 2 h. Subsequently, the hexane was poured off, and the residual hexane was removed by evaporation overnight.

### 3.4. Nutritive Value

All samples were analyzed for their moisture, ash, fat, and protein content according to the Association of Official Agricultural Chemists (AOAC) methods [34]. Carbohydrates were determined by difference by the following formula: 100—(weight in grams (protein + fat + ash + moisture) in 100 g). The conversion method was used to determine the nutritional value [35].

### 3.5. Calculation of Extraction Yield and Efficiency

The extraction yield [23] was calculated as the ratio of the weight of the protein preparation obtained to the weight of the flour used to extract the protein according to the formula:

(1) $$\mathrm{Yield}\;(\%)=\left(\frac{\mathrm{weight}\;\mathrm{of}\;\mathrm{protein}\;\mathrm{preparate}\;\left(g\right)}{\mathrm{weight}\;\mathrm{of}\;\mathrm{flour}\;\left(g\right)}\right)\times 100,$$

The extraction efficiency [23] was calculated as the ratio of the protein content of the resulting protein preparation to the protein content of the insect meal, determined by the Kjeldahl method according to the formula:

(2) $$\mathrm{Extraction}\;\mathrm{efficiency}\;(\%)=\left(\frac{\mathrm{protein}\;\mathrm{content}\;\mathrm{in}\;\mathrm{preparate}\;\left(g\right)}{\mathrm{protein}\;\mathrm{content}\;\mathrm{in}\;\mathrm{insect}\;\mathrm{flour}\;\left(g\right)}\right)\times 100,$$

### 3.6. Functional Properties

#### 3.6.1. Water Holding Capacity

Water holding capacity (WHC) was determined according to the method of Diniz and Martin [36] with a slight modification. The sample (1 g) was mixed with 30 mL of distilled water and stirred with a shaker at 540 rpm for 30 min. Afterwards, the dispersion was centrifuged at 8000 *g* for 15 min. The tubes were placed upside-down on blotting paper (10 min) and weighed. The results were presented as a gram of water absorbed per gram of the sample.

#### 3.6.2. Oil Holding Capacity

The method of Haque and Mozaffar [37] with a slight modification was used to determine oil holding capacity (OHC). The sample (0.5 g) was mixed with 10 mL of vegetable oil and stirred two times for 5 min each with a 10-min break. Afterwards, the dispersion was centrifuged at 8000× *g* for 15 min. The tubes were placed upside-down on blotting paper (10 min) and weighed. The results were presented as a gram of oil absorbed per gram of the sample.

#### 3.6.3. Foaming Properties

Foaming capacity (FC) and foam stability (FS) were determined according to the method of Guo et al. [38]. First, 1 g of the sample and 99 mL of water were homogenized in a high-shear homogenizer mixer (IKA T18 basic, Jarosty, Poland) at a speed of 16,000 rpm for 2 min. The whipped sample was immediately transferred into a cylinder. The total volume was read at time zero and 30 min after homogenization. The foaming capacity and foam stability were calculated from the equations:

(3) $$\mathrm{Foaming}\;\mathrm{capacity}\;(\mathrm{FC})\;(\%)=\left(\frac{V_{0}-V}{V}\right)\times 100,$$

(4) $$\mathrm{Foam}\;\mathrm{stability}\;(\mathrm{FS})\;(\%)=\left(\frac{V_{30}}{V_{0}}\right)\times 100,$$

where V—volume before whipping (mL), V_0_—volume after whipping (mL), V_30_—volume after standing (mL).

#### 3.6.4. Emulsifying Properties

The emulsion activity and emulsion stability were determined according to the method of Wu, Wang, Ma, and Ren [39] with slight modifications. The sample was dispersed in distilled water (1% *w*/*v*), and 10 mL of the dispersion was homogenized (IKA T18 basic, Poland) with 10 mL of vegetable oil at a speed of 16,000 rpm for 1 min. Afterwards, the samples were centrifuged at 3000 *g* for 5 min, and the volume of the individual layers was read. The emulsion stability was evaluated by heating the emulsion for 30 min at 80 °C. Then, the samples were centrifuged at 3000 *g* for 5 min, and the volume of the individual layers was read again. The emulsifying properties were calculated from the equations:

(5) $$\mathrm{Emulsion}\;\mathrm{activity}\;(\mathrm{EA})\;(\%)=\left(\frac{V_{e}}{V}\right)\times 100,$$

(6) $$\mathrm{Emulsion}\;\mathrm{stability}\;(\mathrm{ES})\;(\%)=\left(\frac{V_{30}}{V_{e}}\right)\times 100,$$

where V—total volume of tube contents, Ve—volume of the emulsified layer, V_30_—volume of the emulsified layer after heating.

### 3.7. The Sensory Evaluation

The study was held at the Faculty of Food Science and Biotechnology of the University of Life Sciences in Lublin. Participation in the study was voluntary and was not associated with obtaining compensation. The participants were informed about the usage of the assessment method.

A 5-point rating scale was used to perform the consumer assessment by 55 members, where each note indicates the degree of quality (1—bad, 2—unsatisfactory, 3—satisfactory, 4—good, 5—very good). The characteristics of the flours and protein preparations, such as color, consistency, smell, and overall acceptability, were evaluated. Each of them had a weighting factor (color—0.2, consistency—0.3, smell—0.3, and overall acceptability—0.2) to calculate the product’s overall score.

### 3.8. Statistical Analysis

All assays were performed in triplicate, and the obtained data are presented as means ± SEM (the standard error of the mean). Statistical analyses were carried out using Statistica (version 13.0, StatSoft, Krakow, Poland) for the comparison of means using ANOVA with a post hoc Tukey’s honestly significant difference (HSD) test at the significance level *p* < 0.05.

## 4. Conclusions

The results demonstrate that edible insects could be considered an alternative source of protein and, at the same time, a functional food additive. The defatted flours had the highest protein content of the forms tested. Considering the extraction yield ranged from 32.14% to 51.9% for the studied species, with an extraction efficiency in the range 37.57–56.07%, the process of isolating the protein by the proposed method appears uneconomic due to the lower protein content than in defatted flours. Protein preparations, however, had better functional properties, which is important in the design of food recipes with insect additives. Nevertheless, these properties do not depend on the protein level, but on other factors that create them, such as the amino acid composition of the protein, the presence and distribution of individual amino acid residues, or other factors discussed in the text. As the main objective of introducing insect products into the food industry is to replace conventional food proteins considered expensive or harmful to the environment, further research into optimal insect processing methods is needed to achieve the best compromise between the functionality, taste, and cost-effectiveness, as well as the sustainability and consumer safety, of insect proteins.

## Figures and Tables

**Table 1:** Nutritive value of studied insects form. Different letters in the same column indicate a significant difference (*p* < 0.05).

Species	Form	Protein (%)	Fat (%)	Ash (%)	Carbohydrates (%)	Moisture (%)	Energy Value 100 g	
							kcal	kJ
*Gryllus assimilis*	flour	59.24 ± 0.41 ^e^	26.25 ± 0.45 ^b^	4.05 ± 0.16 ^b^	3.32 ± 0.36 ^f^	7.14 ± 0.21 ^c^	487 ± 7.1 ^b^	2035 ± 21 ^b^
	defatted flour	76.02 ± 0.53 ^a^	3.66 ± 0.52 ^g^	4.93 ± 0.17 ^a^	8.11 ± 0.45 ^c^	7.27 ± 0.48 ^c^	370 ± 5.8 ^e^	1566 ± 12 ^f^
	protein preparation	64.00 ± 0.45 ^d^	12.98 ± 0.86 ^e^	3.88 ± 0.24 ^b^	10.57 ± 0.33 ^a^	7.15 ± 0.69 ^c^	421 ± 4.7 ^d^	1772 ± 13 ^d^
*Acheta domesticus*	flour	64.93 ± 0.45 ^d^	18.54 ± 0.34 ^d^	5.1 ± 0.13 ^a^	4.94 ± 0.21 ^e^	7.29 ± 0.31 ^c^	446 ± 3.4 ^c^	1874 ± 14 ^c^
	defatted flour	75.35 ± 0.53 ^a^	3.43 ± 0.81 ^g^	4.8 ± 0.24 ^a^	9.44 ± 0.55 ^ab^	6.99 ± 0.02 ^c^	370 ± 8.2 ^e^	1568 ± 14 ^f^
	protein preparation	66.34 ± 0.46 ^c^	14.12 ± 1.12 ^e^	3.73 ± 0.07 ^b^	9.31 ± 0.54 ^bc^	6.5 ± 0.05 ^c^	430 ± 9.9 ^cd^	1809 ± 17 ^d^
*Zophobas morio*	flour	49.06 ± 0.34 ^g^	41.9 ± 1.53 ^a^	2.32 ± 0.12 ^c^	3.83 ± 0.33 ^ef^	3.83 ± 0.22 ^d^	585 ± 9.7 ^a^	2433 ± 16 ^a^
	defatted flour	70.51 ± 0.49 ^b^	6.51 ± 0.67 ^f^	3.65 ± 0.35 ^b^	10.57 ± 0.63 ^a^	8.77 ± 0.86 ^b^	383 ± 5.6 ^e^	1619 ± 11 ^e^
	protein preparation	57.35 ± 0.4 ^f^	23.22 ± 1.24 ^c^	2.10 ± 0.11 ^c^	6.14 ± 0.17 ^d^	11.19 ± 0.17 ^a^	447 ± 9.5 ^c^	1870 ± 13 ^c^

**Table 2:** Sensory analysis of edible insect flours and protein preparations thereof. Different letters in the same column indicate a significant difference (*p* < 0.05).

Species	Form	The Studied Characteristics				Overall Score of the Product
		Color	Smell	Consistency	Overall Acceptability	
		Weighting Factor				
		0.2	0.3	0.3	0.2	
*Gryllus assimilis*	flour	4.13 ± 0.72 ^a^	3.38 ± 0.62 ^a^	4.0 ± 0.52 ^ab^	3.75 ± 0.68 ^a^	3.79 ± 0.49 ^ab^
	defatted flour	3.81 ± 0.75 ^a^	4.0 ± 0.89 ^a^	4.38 ± 0.62 ^a^	4.44 ± 0.73 ^a^	4.16 ± 0.51 ^ab^
	protein preparation	4.31 ± 0.48 ^a^	3.25 ± 0.77 ^a^	3.81 ± 0.75 ^ab^	4.13 ± 0.5 ^a^	3.81 ± 0.32 ^ab^
*Acheta domesticus*	flour	4.0 ± 0.89 ^a^	4.38 ± 0.62 ^a^	3.94 ± 0.68 ^ab^	3.88 ± 0.5 ^a^	4.07 ± 0.35 ^ab^
	defatted flour	4.5 ± 0.52 ^a^	4.31 ± 0.87 ^a^	4.63 ± 0.5 ^a^	4.44 ± 0.63 ^a^	4.47 ± 0.47 ^a^
	protein preparation	4.63 ± 0.5 ^a^	3.31 ± 0.95 ^a^	3.63 ± 0.72 ^ab^	4.06 ± 0.68 ^a^	3.82 ± 0.37 ^ab^
*Zophobas morio*	flour	3.13 ± 1.02 ^a^	3.63 ± 0.72 ^a^	2.25 ± 0.68 ^b^	2.69 ± 0.7 ^a^	2.93 ± 0.59 ^b^
	defatted flour	4.19 ± 0.83 ^a^	3.81 ± 0.75 ^a^	4.38 ± 0.72 ^a^	4.25 ± 0.68 ^a^	4.14 ± 0.47 ^ab^
	protein preparation	4.06 ± 0.68 ^a^	3.31 ± 0.6 ^a^	3.88 ± 0.81 ^ab^	3.94 ± 0.68 ^a^	3.76 ± 0.35 ^ab^

## Data Availability

The data presented in this study are available on request from the corresponding author.

## Sample Availability

Samples of the flours and protein preparations from insects are available from the authors.
